# In memory of Univ.-Prof. Dr. med. Normann Willich (1946–2024)

**DOI:** 10.1007/s00066-024-02349-w

**Published:** 2024-12-10

**Authors:** Hans Theodor Eich, Oliver Micke, Michael Baumann, Wilfried Budach, Jürgen Debus, Jürgen Dunst, Rainer Fietkau, Uwe Haverkamp, Mechthild Krause, Franz-Josef Prott, Gabriele Reinartz, Claudia Rübe, Christian Rübe

**Affiliations:** 1 Department of Radiation Oncology, Münster University Hospital, Albert-Schweitzer-Campus 1, 48149 Münster, Germany; 2 Department of Radiotherapy and Radiation Oncology, Franziskus Hospital Bielefeld, 33615 Bielefeld, Germany; 3 German Cancer Research Center (DKFZ), 69120 Heidelberg, Germany; 4 National Center for Tumor Diseases (NCT) Heidelberg, 69120 Heidelberg, Germany; 5 German Cancer Consortium (DKTK), 69120 Heidelberg, Germany; 6 Department of Radiation Oncology, University Hospital Düsseldorf, 40225 Düsseldorf, Germany; 7 Department of Radiation Oncology and Radiotherapy, University Hospital Heidelberg, 69120 Heidelberg, Germany; 8 Department of Radiation Oncology, University Hospital Schleswig-Holstein, Campus Kiel, 24105 Kiel, Germany; 9 Department of Radiation Oncology, University Hospital Erlangen, 91054 Erlangen, Germany; 10 Department of Radiation Oncology, University Hospital Carl Gustav Carus, 01307 Dresden, Germany; 11 RNS Gemeinschaftspraxis Strahlentherapie, 65189 Wiesbaden, Germany; 12 Department of Radiation Oncology, University Hospital Saarland, 66421 Homburg/Saar, Germany

**Fig. 1:**
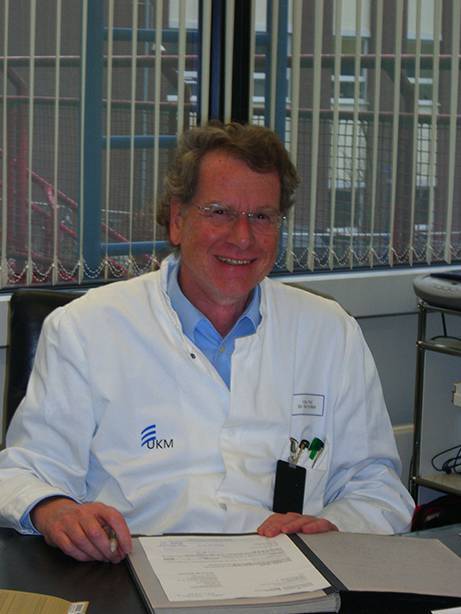
Prof. Dr. Normann Willich

Prof. Dr. Normann Willich (Fig. 1), the former Director of the Department of Radiation Oncology of Muenster University Hospital, passed away on September 10, 2024, at the age of 78. His death marks the loss of one of the most influential figures in German radiation oncology.

Normann Willich was born in Oldenburg, Lower Saxony, on June 22, 1946. He began his medical studies at the University of Kiel in 1967 and completed his degree in 1974. He subsequently rotated through a diverse number of fields as a first-year intern, including pediatrics, neurology/psychiatry, anesthesiology, internal medicine, and surgery in Berlin, Oberhausen, Kiel, and Augsburg.

In 1976, during his surgical and radiology training in Augsburg, Prof. Willich encountered the field of radiation oncology, a moment that would dramatically alter the course of his medical career. He pursued further specialization at the Munich University Hospital, where he completed his doctorate in 1981 and his habilitation in 1989. His habilitation thesis evaluated the use of hyperthermia in malignant tumors, though he would later go on to work in a diverse set of other scientific areas as well. In 1991, Prof. Willich was appointed full professor and Director of the Department of Radiation Oncology at the University of Münster. For 20 years, until his retirement in 2011, he shaped the department with his vision. He also temporarily served as Acting Director of Radiology following the passing of Prof. Peters, demonstrating both his leadership and the recognition he received within the institution.

Over the course of his professional career, Prof. Willich was a prolific researcher, authoring and coauthoring more than 250 publications in peer-reviewed journals and more than 100 book chapters in renowned textbooks. He performed valuable pioneering work on intraoperative radiotherapy in pancreatic and rectal cancer, in malignant brain tumors, and in breast cancer, using electron beams or HDR brachytherapy. He also helped to assess novel treatment paradigms in gastrointestinal lymphoma therapy, establishing radiotherapy as the first-line treatment in the majority of cases. He later provided much-needed prospective multicenter data to improve the safety and efficacy of pediatric cancer treatments via his involvement in the RiSK study (Late Effects after Radiotherapy in Childhood and Adolescence).

As a dedicated mentor, Prof. Willich supervised more than 50 doctoral students and 10 habilitations, and many of those he supervised have gone on to play leading roles in radiation oncology. Students and colleagues alike admired him for his collaborative spirit, his intellectual rigor, and his unwavering dedication to scientific excellence.

He was also an active and influential member of numerous professional societies:

He was President of the German Society of Radiation Oncology (DEGRO) from 2005 to 2007 and served as secretary general of the DEGRO for 10 years from 2011 to 2021. In addition, he was a board member of the European Society for Therapeutic Radiation Oncology (ESTRO), the German Hodgkin’s Lymphoma Study Group, and the German Society of Pediatric Oncology and Hematology (GPOH). He was also selected as a chairman of the German Working Group of Pediatric Radiation Oncology (APRO) and the German Study Group of Radiation-Induced Late Effects in Children. Furthermore, he was a member of the German Cancer Society (*Deutsche Krebsgesellschaft*), the International Society of Intraoperative Radiation Oncology (ISIORT), and the Pediatric Radiation Oncology Society (PROS), and was a reference Radiation Oncologist for the German Pediatric AML Study Group, the German Gastrointestinal Lymphoma Study Group, and the German Thyroid Cancer Study Group. He was a board member of the Professional Association of German Radiation Oncologists (BVDST) from 2009 to 2011 and Vice Chairman of the Medical Authority according to StrSchV (*Ärztliche Stelle der Ärztekammer*) Westfalen-Lippe from 2003 to 2011. He also served as a coeditor of the renowned German radiation oncology journal *Strahlentherapie und Onkologie.*

In recognition of his achievements for German radiation oncology, he was elected Honorary Member of the German Society of Radiation Oncology (DEGRO) in 2016.

Prof. Willich was also highly involved in the administration of the University of Münster: he was a member of the senate from 1995 to 1998 and from 2007 to 2009, Vice Dean of the Faculty of Medicine from 1997 to 1998, and Vice Rector for Research and Young Scientists from 1998 to 2002.

He carried out all the responsibilities he took on with enormous enthusiasm and great care for the cause.

**Fig. 2:**
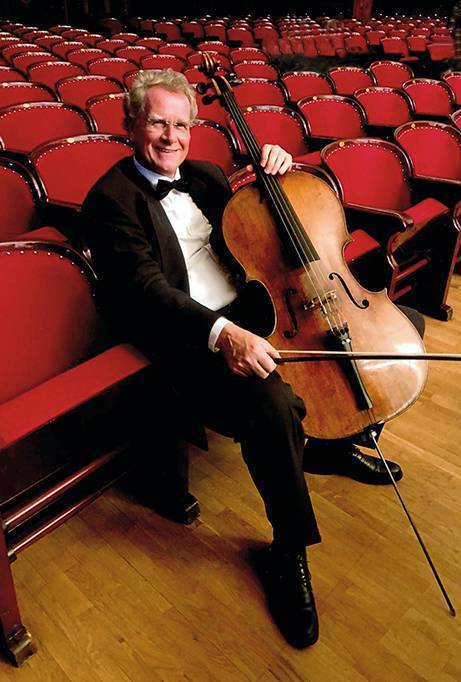
Prof. Dr. Normann Willich, a highly skilled cellist

On a personal level, Normann Willich was a true North German at heart—stubborn at times, but always even-tempered and deeply compassionate. He had a gift for balancing his demanding professional life with personal passions, most notably his love of music. A highly skilled cellist, he found fulfillment playing in orchestras such as the Management Symphony Leipzig, the Alte Philharmonie Münster, and the World Doctors Orchestra. Music provided him with both relaxation and joy, helping him to maintain perspective amidst the pressures of his work (Fig. 2). Prof. Willich also had a profound interest in art and culture, especially modern paintings. His intellectual curiosity extended far beyond the realm of medicine, and he was a fascinating conversationalist, always ready to engage in lively discussions on a wide range of topics.

With the passing of Professor Willich, we have lost one of the most committed and consistent advocates of radiation oncology, particularly among the German-speaking countries. While his contributions were centered at Münster University Hospital, his influence was felt nationally and internationally. His legacy will live on through the many lives he touched—both in his scientific endeavors and with his deeply humane approach to patient care.

Normann Willich is survived by his wife, Regina Rentschler-Willich, and their daughter, Dr. Caroline Willich. Our deepest condolences go out to them during this difficult time.

We will always remember his words on how to get a good balance in life: through the right dosage of stress (but without sport!) and ending the day with a glass of red wine. We pay tribute to his monumental achievements and cherish the remarkable legacy he has left behind.

